# A Humanized Monoclonal Antibody Specific for Invariant Natural Killer T (iNKT) Cells for *In Vivo* Depletion

**DOI:** 10.1371/journal.pone.0076692

**Published:** 2013-09-27

**Authors:** Felix Scheuplein, Abraham Thariath, Susan Macdonald, Alemseged Truneh, Robert Mashal, Robert Schaub

**Affiliations:** NKT Therapeutics, Inc., Waltham, Massachusetts, United States of America; La Jolla Institute for Allergy and Immunology, United States of America

## Abstract

Invariant Natural Killer T (iNKT) cells are a subset of T cells recognizing glycolipid antigens presented by CD1d. Human iNKT cells express a conserved T cell receptor (TCR)-α chain (Vα24-Jα18) paired with a specific beta chain, Vβ11. The cells are both innate-like, with rapid cytokine release, and adaptive-like, including thymic positive selection. Over activation of iNKT cells can mediate tissue injury and inflammation in multiple organ systems and play a role in mediating the pathology associated with clinically important inflammatory diseases. At the same time, iNKT cell activation can play a role in protecting against infectious disease and cancer or modulate certain autoimmune diseases through its impact on both the innate and adaptive immune system. This suggests that approaches to cause iNKT cell reduction and/or depletion could treat inflammatory diseases while approaches to promote activation may have therapeutic potential in certain infections, cancer or autoimmune disease. This report summarizes the characterization of a humanized monoclonal depleting antibody (NKTT120) in the cynomolgus macaque. NKTT120 is being developed to treat iNKT mediated inflammation that is associated with chronic inflammatory conditions like sickle cell disease and asthma. NKTT120 binds to human iTCRs and to FCγRI and FCγRIII and has been shown to kill target cells in an ADCC assay at low concentrations consistent with the FCγR binding. iNKT cells were depleted within 24 hours in cynomolgus macaques, but T cell, B cell, and NK cell frequencies were unchanged. iNKT cell recovery was dose and time dependent. T cell dependent antigen responses were not impaired by NKTT120 mediated iNKT depletion as measured by response to KLH challenge. NKTT120 administration did not induce an inflammatory cytokine release at doses up to 10 mg/kg. These data support the use of NKTT120 as an intervention in inflammatory diseases where iNKT reduction or depletion could be beneficial.

## Introduction

Natural killer T (NKT) cells are a subset of T lymphocytes that share surface markers and functional characteristics with both conventional T cells and natural killer (NK) cells [1]. NKT cells recognize glycolipid antigens rather than peptide antigens presented on the major histocompatibility complex (MHC)-I-like protein CD1d, expressed on the surface of antigen presenting cells [2]. In addition, while most T cell subpopulations have diverse sequences for their T Cell Receptors (TCRs), Type 1 NKT cells, express a uniquely rearranged, highly conserved, invariant TCR-α chain (Vα24-Jα18 in humans), which preferentially pairs with specific TCR-β chains (Vβ11 in humans). This receptor, the invariant T cell receptor (iTCR) is identical across individuals and these cells are known as invariant NKT cells (iNKT). iNKT cells represent a very small subset of the total T cell population in human and non-human peripheral blood. In humans, they range from less than 0.01% of all T cells to higher than 1.0%, with the majority of individuals clustering at the lower end of the range [3,4]. The iNKT cell shares characteristics of both the innate and adaptive arms of the immune system and thus play a unique role by modulating T and B cell responses as well as innate immunity [5]. Like cells of the innate system, iNKT cells are rapid-onset cells with a universal receptor, but they can also be placed squarely in the adaptive system because they share other properties of T lymphocytes. As such, they serve as a bridge between the two systems where they can play either a pro-inflammatory or an immuno-regulatory role [1].

iNKT cells have been shown to be involved in both a protective role in regard to infection, cancer and certain autoimmune diseases and in mediating tissue injury and inflammation in multiple organ systems, including liver, kidney, skin, lung, heart, intestine and spinal cord in some chronic inflammatory diseases such as asthma and sickle cell disease [6–8]. These diverse actions of iNKT cells suggest that manipulation of iNKT cell function could be an effective treatment with activation or depletion approaches dependent on the contribution of the iNKT cell status in a specific disease. The ability to study the role of iNKT cells in human disease has been limited by the lack of available agents that permit therapeutic manipulation and chronic treatment. In regard to the treatment of iNKT mediated chronic inflammatory diseases, we have developed a humanized depleting antibody (NKTT120) that is specific to the human invariant T cell receptor and the receptor of certain non-human primate iNKT cells. NKTT120 is being developed to evaluate its ability to reduce inflammation associated with sickle cell disease and moderate/severe asthma, two conditions where iNKT cell activation has been shown to be involved in the inflammatory state associated with these conditions [1,5,6,9–12]. The purpose of this report is to review the non-clinical characterization of this antibody to support its clinical use both in vitro and in vivo using the non-human primate to determine its PK, effective depleting doses and specificity for iNKT cells. The results of these studies demonstrate that NKTT120 is highly specific and effective in mediating the depletion of iNKT cells in vivo, and they support the clinical evaluation of this antibody in the treatment of both acute and chronic inflammatory diseases.

## Materials and Methods

### In Vitro Studies

#### Generation of humanized ant-iTCR antibody

A synthetic cyclic peptide modeled after the junctional sequence of Vα24-Jα18 of the invariant human T cell receptor, was covalently linked to a carrier protein to immunize CD1d knockout mice, which lack iNKT cells. Hybridomas from the spleen of these mice were screened for iTCR specific antibodies and the monoclonal antibody 6B11was identified [13].

The variable region of the murine 6B11 was humanized using Composite Human Antibody™ technology (Antitope Ltd, Cambridge, UK). One antibody construct (NKTT120, an IgG1κ) was selected from 48 generated based on their affinity to the human iTCR, productivity and purity analyses. Its sequence was used to construct the expression vector for production. A dicistronic vector with puromycin resistance was constructed (Selexis SA, Geneva, Switzerland) and transfected by microporation into a Chinese Hamster Ovary (CHO) cell line. A clone and subclone were selected based on growth and productivity. Purified study material was produced in a 50L single use fermenter.

#### Affinity Characterization of NKTT120

Binding analysis of NKTT120 was performed by surface plasmon resonance on a Biacore 3000 instrument (Biacore AB, Uppsala, Sweden; Blue Stream Laboratories, Cambridge MA, USA). NKTT120 was immobilized on the chip to study its interaction with FcγRI, FcγRIIIa and C1q. Interaction analysis was performed at six different analyte concentrations to derive the kinetic and thermodynamic parameters. The curves generated from the interaction analysis was plotted using BiaEvaluation software (Biacore AB, Uppsala, Sweden) to determine the dissociation constant K_D_.

#### Cytotoxicity activity of NKTT120

Antibody dependent cellular cytotoxicity (ADCC) assays were performed using a cell line expressing recombinant iTCR on the cell surface as the target cells (T) and CD16-transduced NK-92 high affinity receptor cells ([CST-103] Konkwest, Del Mar, CA) as the effecter cells (E). Assays were performed at a 60:1 E:T ratio in the presence of NKTT120 at either 1 µg/mL or 20 µg/mL. NK-92 cells not transduced with CD16 were used as the experimental control and nonspecific IgG1 was used as the negative control. Target cells were Calcein AM loaded and the percent cytotoxicity was calculated based on fluorescence release (Antitope LTD, Cambridge, UK).

CDC assays were performed using 5 x 10^5^ cells/mL iTCR expressing recombinant cell line as target cells in a 96 well plate. Antibody was added to the plate at 2, 20 and 100 µg/mL final concentration. Human serum complement at a final concentration of 13% was added and the plate was incubated for 3 hours at 37 °C. The plate was incubated for 10 minutes in the dark and then read in a Fluostar optima plate reader. An irrelevant IgG1 was used as the negative control. Anti CD-52 antibody with Reh cells (ATCC cat. # CRL-8286) were included as positive control for the assay. CDC activity was calculated as %CDC (Antitope LTD, Cambridge, UK).

#### Flow Cytometry

Flow Cytometry to monitor iNKT cells and assess specificity of NKTT120 was performed on heparinized human, NHP and rodent peripheral blood samples. Briefly, whole-blood samples were stained with fluorochrome labeled monoclonal antibodies for 30 min at 4°C, washed, red blood cells were lysed with 1 x RBC lysis buffer (Biolegend, San Diego, CA), washed again and then analyzed by flow cytometry. For binding specificity, human cell lines (Jurkat, Raji, THP-1, U937, K562, HEK293, HeLa, FO-1 and LNCap, kindly provided by Dr. Steven Balk, BIDMC, Boston MA) were incubated with NKTT120, isotype control or anti Human MHC-1, respectively, washed, incubated with secondary antihuman IgG mAb, washed and analyzed by flow cytometry (BD LSR Fortessa 12 color flow cytometer, BD Biosciences, San Jose, CA; Flowjo 9.5 Software, Treestar, Ashland, Oregon).

### In Vivo Studies

#### Ethics Statement

All in vivo primate studies were performed at MPI Research. All studies were in compliance with the US Department of Agriculture’s Animal Welfare Act (9 CFR Part 1, 2 and 3) and the Guide for the Care and Use of Laboratory Animals, Institute of Laboratory Animals Resources, Washington, D.C. under a protocol that was reviewed and approved by the Institutional Animal Care and Use Committee at MPI Research, Mattawan, MI (PHS/OLAW Assur. #: A3181-01 ; AAALAC File #: 000243; USDA Reg. #: 34-R-0031). 

The standard non-human primate housing was Group 3 primate caging (connected cages) with each individual cage being 26.5” wide x 23.5” deep and 31” height, which is considered adequate for monkeys up to 10 kg. The cages have perches and automatic watering. All primates are provided a bowl and a mirror as well as additional enrichment items that included special foods (fruits, vegetables, special treats) and visual/auditory stimulation (movies, music etc). Group housed animals were same sex and from the same treatment group. Animals were housed separately for urine collection or if unusually aggressive with cage mates. The light cycle was fluorescent lighting for approximately 12 hours per day. Temperature and humidity were continuously monitored, recorded and maintained to a maximum extent possible within the protocol-designated ranges of 64 to 84 degrees F and 30-70%, respectively. Lab Diet (Certified Primate Diet #5048, PMI Nutrition International, Inc.) was available twice a day and other enrichment foods were provided on a regular basis. The pain/distress classification of the study was USDA Category D, having the potential for more than momentary or slight pain (including unknown toxicity. Under this category pain or discomfort will be relieved by anaesthetics, analgesics, veterinary care or euthanasia if necessary. Some animals in this study were returned to the colony following treatment and evaluation. Some animals were euthanized as required to allow complete gross and microscopic tissue examination for toxicologic assessment. These animals were administered euthanasia solution under ketamine sedation if necessary followed by a research standard operating procedure approved method to ensure death (e.g. exsanguination). All procedures were reviewed and approved the Institutional Animal Care and Use committee before animal receipt or transfer. Animal housing, care and treatment was in compliance with the US Department of Agriculture’s Animal Welfare Act (9 CFR Parts 1, 2, and 3) and The Guide for the Care and Use of Laboratory Animals, Institute of Laboratory Animal Resources, National Academy Press, Washington, D.C., 2011. The facility also maintained an Animal Welfare Assurance statement with the National Institutes of Health, Office of Laboratory Animal Welfare.

Forty-four (16 male, 28 female) cynomolgus monkeys (Maccaca *fascicularis*; MPI Research Mattawan, MI) were evaluated.

#### Depletion /Reappearance Study

Twelve of the female cynomolgus monkeys (2-5 years old, 2-5 kg) were used to explore the relationship between dose and duration of iNKT cell depletion. Each group of animals (n=3) received a single dose of NKTT120. The dosing groups were 10, 30, 100 and 300 µg/kg respectively. iNKT cells and other lymphocytes were monitored 24, 48, 72, 96 and 168 hours following dosing and weekly thereafter with heparinized whole blood using flow cytometry (BD Fortessa flow cytometer with FACSDIVA software, BD Biosciences, San Jose, CA). Serum samples for pharmacokinetics were collected at 1, 6, 24, 48, 72, 96, 168, 336, 504 and 672 hours following dosing, and every other week. The data presented here represent 106 days post treatment sampling. A non-compartmental module using (WinNonlin version 6.3, Pharsight Corp, St. Louis MO) was used for analysis. One way ANOVA with multiple comparison followed by a non-parametric T test was performed using PRISM software on all the subsets.

#### Repeat Dose Study

A repeat dose study was conducted to evaluate the potential subchronic toxicity of NKTT120. Three treatment groups of the male and female cynomolgus monkeys (2-5 years of age and 2-5 kg) were administered the test article weekly at respective dose levels of 0.3 (n=3M; 3F), 3 (n=3M; 3F), or 10 (n=5M; 5F) mg/kg for five consecutive weeks. One additional group of animals (n=5M; 5F) served as the control and received the vehicle. The test article or vehicle was administered to respective groups via slow bolus intravenous injection at a dose volume of 1.0 mL/kg. Following the last dose administration period on Day 29, all animals were euthanized and necropsied with the exception of two animals/sex from the 0 and 10 mg/kg dose groups. These animals were maintained and observed for an 8-week treatment-free recovery period. Blood samples were collected for immunophenotyping pretest (Week -1), at 24, 48 and 96 hours following the first dose, at 24 hours following each subsequent dose and once every other week thereafter, including once prior to termination. One way ANOVA with multiple comparison followed by a non-parametric T test was performed using PRISM software on all groups.

## Results

### Biologic and biophysical characterization of NKTT120

**Figure 1:**
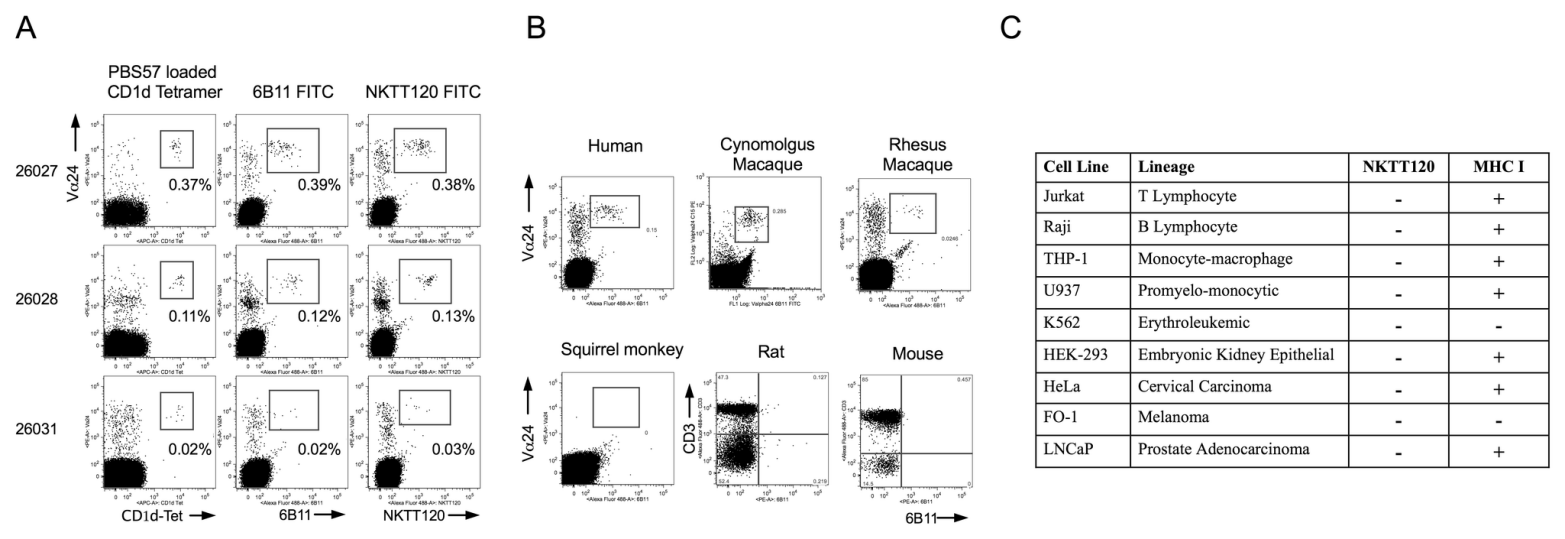
Whole blood samples from healthy volunteers were stained with antibodies against CD3 (Clone SP34.2), TCR Vα24 (Clone C15) and either PBS57 loaded CD1d Tetramer, 6B11 or NKTT120. Red blood cells were subsequently lysed, cells washed and analyzed by flow cytometry. The data shown is gated on CD3+ cells (**A**). Human, Cynomolgus Macaque, Rhesus Macaque, Squirrel Monkey whole blood samples and rat and mouse splenocytes were stained with antibodies against CD3 (Primates: Clone SP34.2, Rat: Clone 1F4, mouse: Cone 17A2), TCR Vα24 (Clone C15) and invariant TCR (6B11). Red blood cells were subsequently lysed, cells washed and analyzed by flow cytometry (B). Human cell lines (106 per sample) were incubated with NKTT120 or isotype control, washed and stained with anti Human IgG (Clone G18-145) and pan MHC-I (Clone G46-2.6), washed and analyzed by flow cytometry (C).

Flow-cytometric binding analyses demonstrated that NKTT120, similarly to its parent molecule 6B11 and to PBS57 loaded CD1d-tetramer, specifically binds to the invariant TCR on human peripheral blood iNKT cells (Figure **1A**). While 6B11 and NKTT120 cross-react with iNKT cells from old-world non-human Primates, NKTT120 does not cross-react with new world non-human Primate species or rodent iNKT cells (Figure **1B**). To further corroborate specificity of NKTT120 for iNKT cells, flow based binding analyses were performed using a panel of human hematopoietic and non-hematopoietic cell lines. While the positive control, pan MHC-I antibody, bound to all cell lines expressing MHC-I, NKTT120 did not bind to any non-iNKT cell line tested (Figure **1C**). To characterize the biophysical properties of NKTT120 Surface Plasmon Resonance analyses were used to determine the binding affinity to recombinant human invariant TCR, FCγRI, FCγRIII and C1q. The data are summarized in Table **1**. The binding affinity of NKTT120 to the human iTCR was determined to be 44 nM. FCγRI, FCγRIII, as expected for an IgG1κ monoclonal antibody, bound NKTT120 with affinities of 12 nM and 122 nM, respectively. While some low binding interaction to C1q was detectable at very high concentrations, the data did not permit a K_D_ determination. To determine the possible mode of action for NKTT120 mediated iNKT cell depletion we established ADCC and CDC assays using stable HEK293 invariant TCR transfectants. Targets were efficiently killed by a NK cell-line in the presence of low concentrations of NKTT120. Consistent with the Biacore data that showed only weak C1q binding, no killing is observed at low antibody concentrations, but NKTT120 specific CDC can be observed at very high concentrations (Table **1**).

**Table 1:** Summary of biophysical characteristics of NKTT120.

Biacore affinity	
**NKTT120 interaction partner**	**K_D_**
Hu iTCR	44 nM
FcγRI	12 nM
FcγRIII	122 nM
C1q	Minimal interaction, K_D_ not determinable
ADCC	
**NKTT120 [µg/ml]**	**(Cell killing)**
1	34%
20	44%
CDC	
**NKTT120 [µg/ml]**	**(Cell killing)**
1	BLQ
20	20%
100	41%

### Single Dose Recovery study

A single-dose depletion/recovery study allowed us to evaluate the pharmacokinetics of NKTT120 and to better understand the kinetics of NKTT120 mediated depletion of iNKT cells and their recovery.

**Figure 2:**
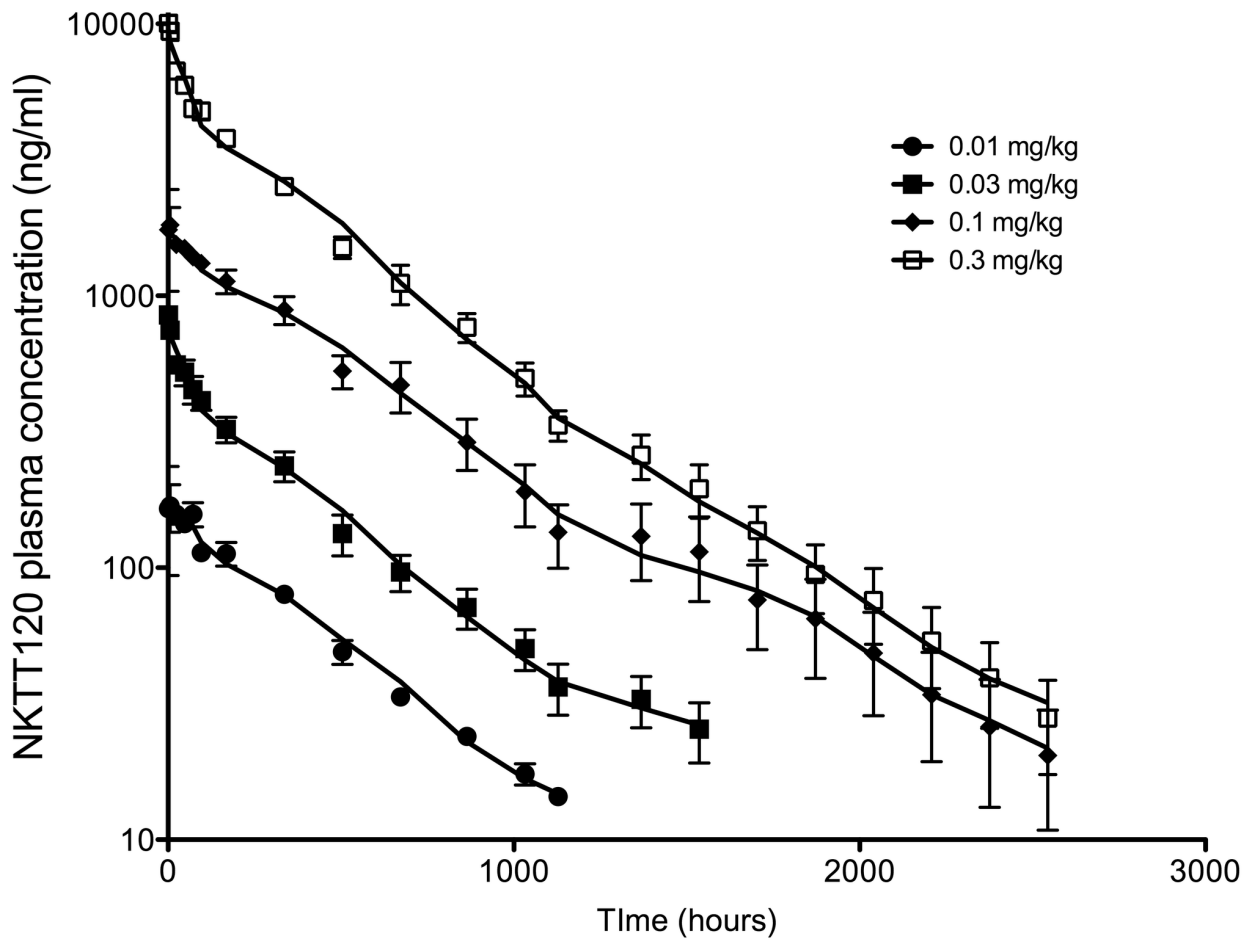
Cynomolgus macaques (n=3 per dose group) were dosed with a single dose of 0.01 mg/kg (filled circles), 0.03 mg/kg (filled squares), 0.1 mg/kg (filled diamonds) and 0.3 mg/kg (open squares) of NKTT120, respectively and subsequently bled at indicated time points. NKTT120 plasma concentrations were measured by ELISA. Shown are means within groups with standard error.

Key pharmacokinetic parameters are summarized in Table **2**. The mean clearance of NKTT120 was 0.131 mL/hr/kg across doses. The average volume of distribution at steady state (Vss) was 63.7 mL/kg. The apparent terminal half life was 420 hours (17.5 days). Concentrations of NKTT120 decline in a bi-exponential manner and in parallel during the apparent terminal phase following intravenous administration (Figure **2**).

**Table 2:** Summary of pharmacokinetic properties of NKTT120 in cynomolgus macaques.

Dose (mg/kg)		Clearance (mL/h/kg)	Half Life (h)	C_max_ (ng/mL)	Vss (mL/kg)	AUC (h*ng/mL)
0.01 (n=3)	Mean	0.144	332	236	67.1	69680
	SD	0.00188	48.9	0	4.35	911
0.03 (n=3)	Mean	0.131	569	851	72.1	239784
	SD	0.0353	172	109	13.2	58151
0.1 (n=3)	Mean	0.132	397	2460	65.8	809505
	SD	0.0475	87.7	141	1.36	291208
0.3 (n=3)	Mean	0.121	344	10030	51.8	2511616
	SD	0.0182	63.5	1272	3.5	383868

**Figure 3:**
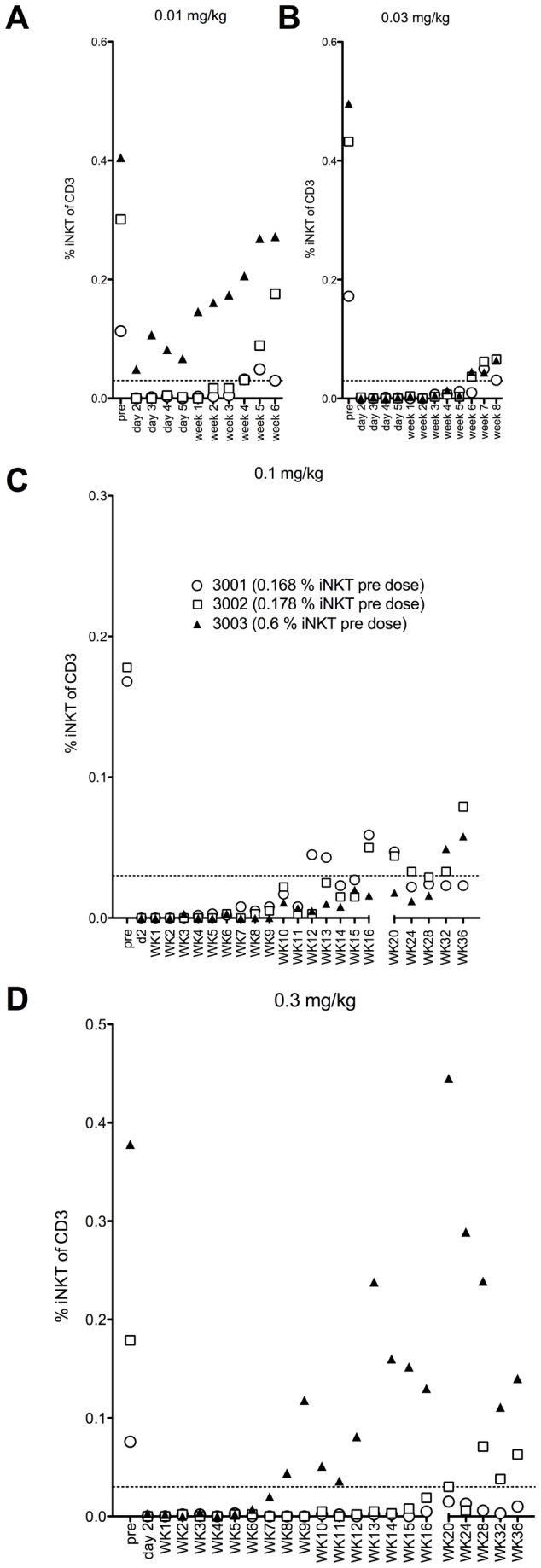
Cynomolgus macaques (n=3 per dose group) were dosed with a single dose of 0.01 mg/kg (A), 0.03 mg/kg (B), 0.1 mg/kg (C) and 0.3 mg/kg (D) of NKTT120, respectively and subsequently bled at indicated time points. Whole blood samples were stained with antibodies against CD3 (SP34.2), CD20 (2H7), TCR Vα24 (C15), CD159a (Z199) and invariant TCR (6B11). Subsequently red blood cells lysed, cells washed and analyzed by flow cytometry. iNKT cells were identified as CD3+, Vα24+, 6B11+ lymphocytes and reported as % of CD3+ cells.

**Figure 4:**
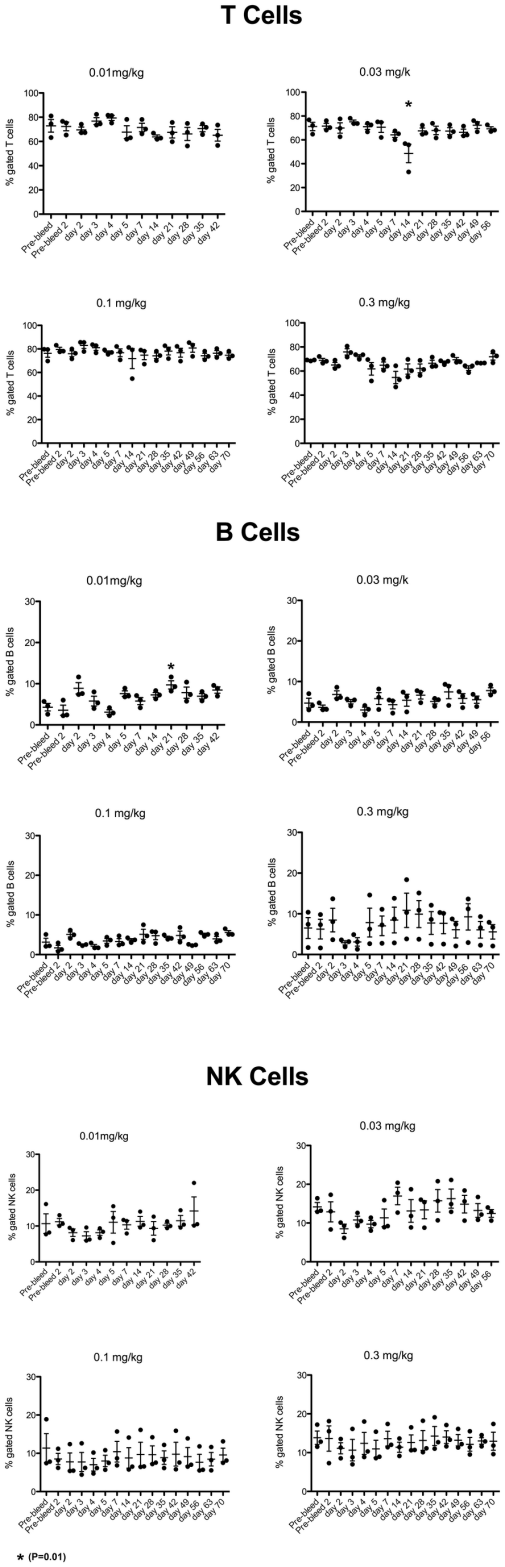
Cynomolgus macaques (n=3 per dose group) were dosed with a single dose of 0.01 mg/kg, 0.03 mg/kg, 0.1 mg/kg and 0.3 mg/kg of NKTT120, respectively and subsequently bled at indicated time points. Whole blood samples were stained with antibodies against CD3 (SP34.2), CD20 (2H7), TCR Vα24 (C15), CD159a (Z199) and invariant TCR (6B11). Subsequently red blood cells lysed, cells washed and analyzed by flow cytometry. T cells were identified as CD20-, CD3+ and 6B11- lymphocytes, B cells were identified as CD3-, CD20+ lymphocytes and NK cells were identified as CD3-CD159a+ lymphocytes. T, B and NK cells are reported as % of lymphocytes One way ANOVA with multiple comparison followed by a non-parametric T test was performed using PRISM software on all the subsets. * indicates significant difference to pre-bleed values (P=0.01).

**Figure 5:**
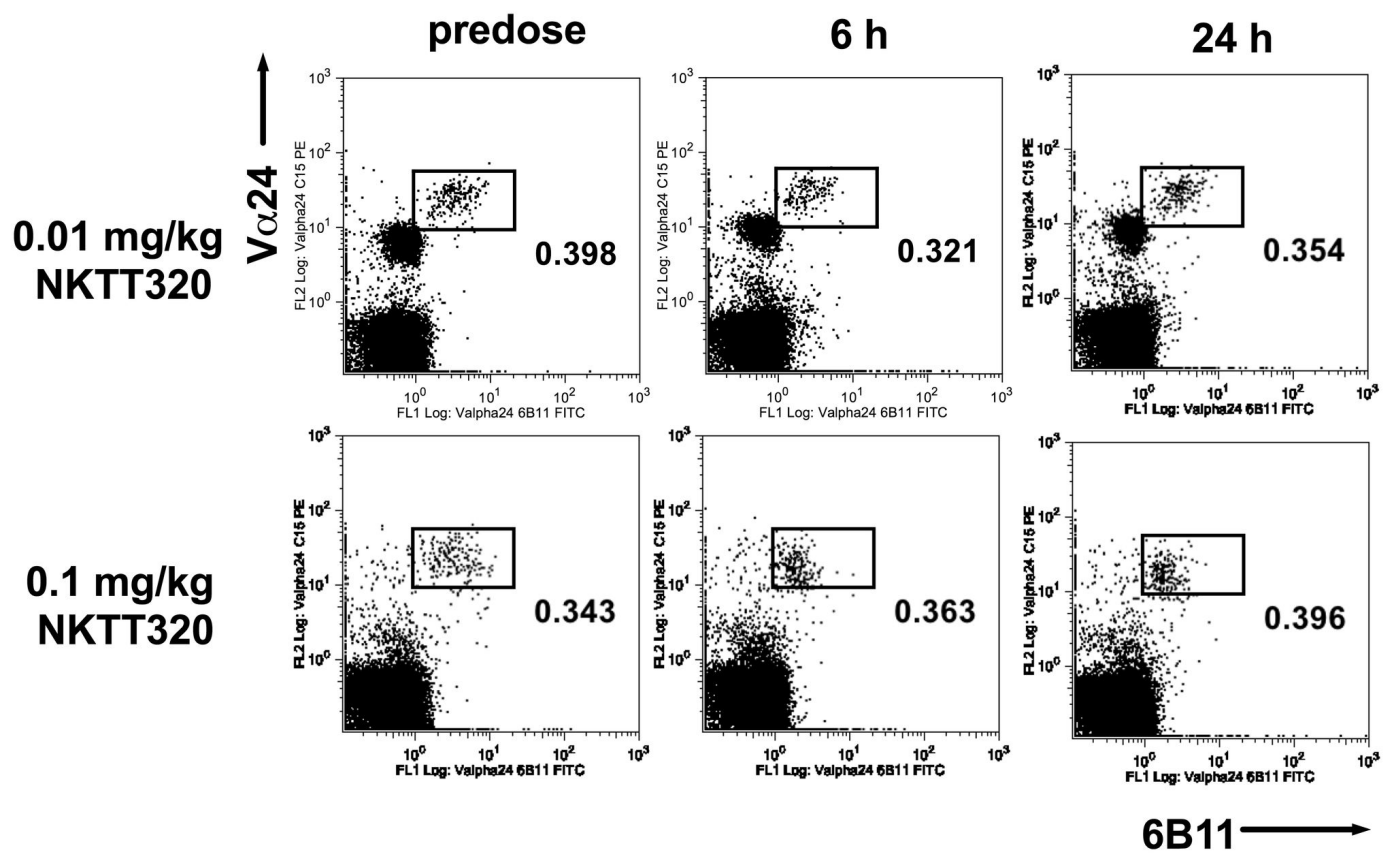
Cynomolgus macaques in a dose-range finding study (n=1 per dose) were dosed with a single dose of 0.01 mg/kg and 0.1 mg/kg NKTT320, respectively and subsequently bled at indicated timepoints. Whole blood samples were stained with antibodies against CD3 (SP34.2), CD20 (2H7), TCR Vα24 (C15), CD159a (Z199) and invariant TCR (6B11). Red blood cells were subsequenltly lysed, cells washed and analyzed by flow cytometry. iNKT cells were identified as CD3+, Vα24+ and 6B11+ lymphocytes and reported as % of CD3+ cells.

iNKT cells were depleted within 24 hours of dosing in all groups (Figure **3**), with the exception of one animal in the 0.01 mg/kg group that received the dose by subcutaneous injection (as judged by the concentration-time plot). This animal’s iNKT cell number was reduced by ~85%. The maximum plasma concentration following these doses was ≥ 200 ng/mL. There was no change in other cells of the lymphocytic series in any dose group (Figure **4**). Time to reappearance was then monitored. Reappearance was defined as a return of iNKT cells to ≥0.03% of CD3+ cells in all animals of a group for two consecutive weeks. This corresponds to the average iNKT cell number in the subset of Cynomolgus macaques that had measurable iNKT cells in the pre-screen portion of the study (25% of females screened), with 0.01% of CD3+ cells being defined as the lower limit of quantification. The duration of iNKT cell depletion was dose dependent. The 0.01 mg/kg dose group recovered at Week 5 and the 0.03 mg/kg dose group recovered at Week 8 (Figure **3 A, B**). In the 0.1 mg/kg dose group animals began to show measurable iNKT cells at week 10. From week 32 all animals in the group had reached the recovery criterion at one point during the study, but not at the same time, so recovery was not achieved for the group (Figure **3C**). In the higher dose group with the exception of one animal that showed recovery from week 8, animals started showing measurable iNKT cells by week 16 throughout the remainder of the study with 3/6 demonstrating recovery to 0.03% (Figure **3D**). The concentration of NKTT120 that was associated with the return of measurable peripheral iNKT cell counts was ≤ ~60 ng/mL. Recovery to 0.03%, when it occurred, was associated with a plasma concentration of ~30 ng/mL. No adverse events were observed in this study. This in vivo depletion is consistent with the in vitro ADCC and CDC results. The possibility that the decline in iNKT cell numbers was caused by epitope masking, receptor internalization or iNKT cell marginalization into from peripheral blood into tissues was addressed in a small study that used NKTT320, an antibody that shares the variable regions with NKTT120, but has a mutated IgG4 backbone that does not support Fc-receptors or C1q binding and thus not induce CDC/ADCC. A dose of 0.1 mg/kg NKTT320 did not induce a change in iNKT cell numbers (Figure **5**). These NKTT320 data indicate that from the 0.01 mg/kg NKTT120 dose upwards iNKT cells were truly depleted and that lack of iNKT cell detection was not due to epitope masking or receptor internalization.

#### Repeat Dose study

**Figure 6:**
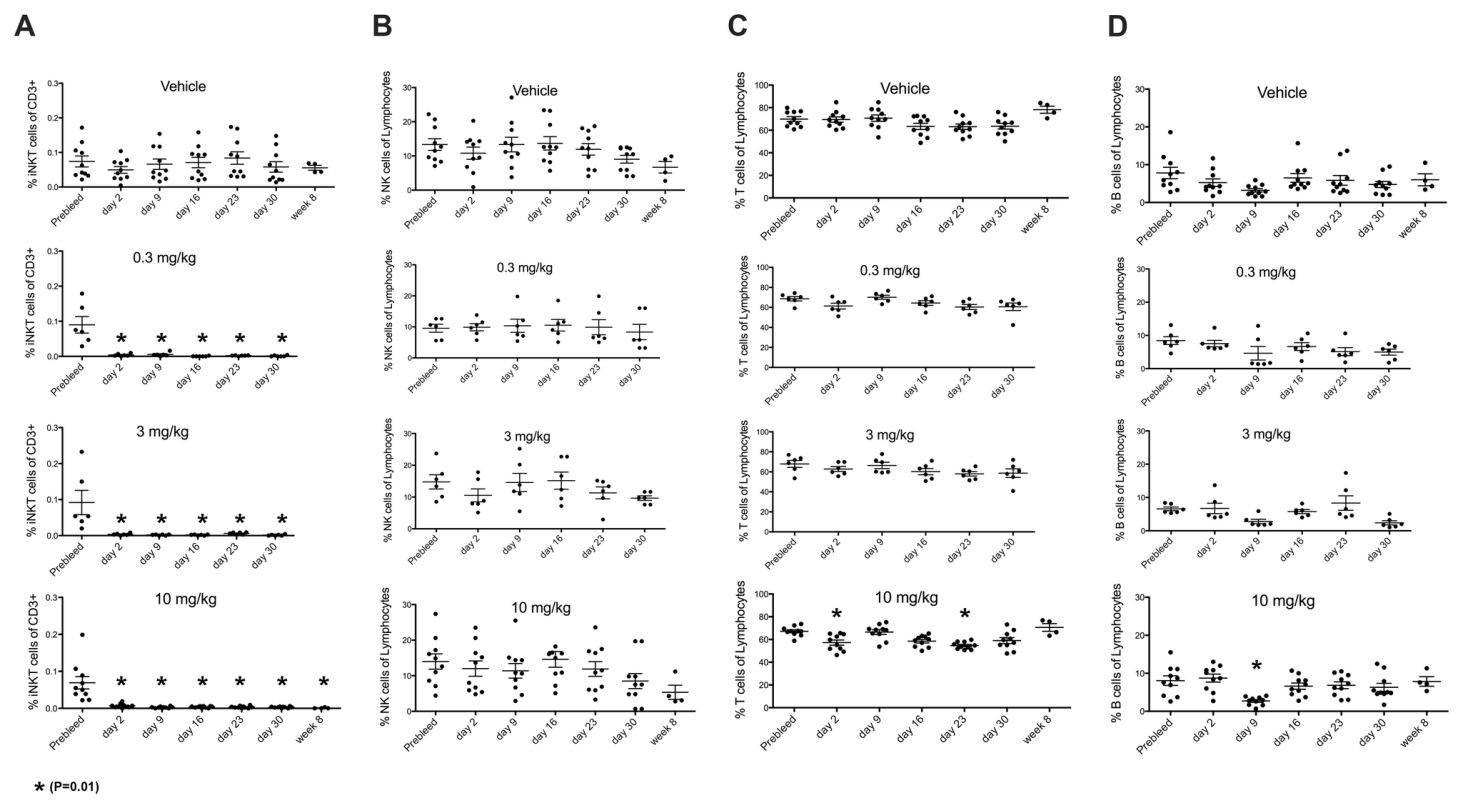
Cynomolgus macaques (n = 5 females + 5 males per group) were dosed and then weekly redosed for 4 weeks (total of 5 doses) with the indicated doses of NKTT120. Whole blood samples were drawn at indicated timepoints and stained with antibodies against CD3 (SP34.2), CD20 (2H7), TCR Vα24 (C15), CD159a (Z199) and invariant TCR (6B11). Red blood cells were subsequenltly lysed, cells washed and analyzed by flow cytometry. iNKT cells were cells identified as CD3+, Vα24+ and 6B11+ lymphocytes and reported as % of CD3+ cells (**A**). NK cells were identified as CD3-, CD159a+ lymphocytes and reported as % of lymphocytes (**B**). T cells were identified as CD3+, CD20- and 6B11- lymphocytes and reported as % of lymphocytes (**C**). B cells were identified as CD3-, CD20+ lymphocytes and reported as % of lymphocytes (**D**). One way ANOVA with multiple comparison followed by a non-parametric T test was performed using PRISM software on all the subsets. * indicates significant difference to pre-bleed values (P=0.01).

iNKT cells were depleted within 24 hours in all dose groups and stayed fully depleted for the duration of the study. Figure **6** demonstrates the specificity of NKTT120 for targeting iNKT cells. While iNKT cells are completely depleted, no changes in T cell, B cell or NK cell numbers were observed.

**Figure 7:**
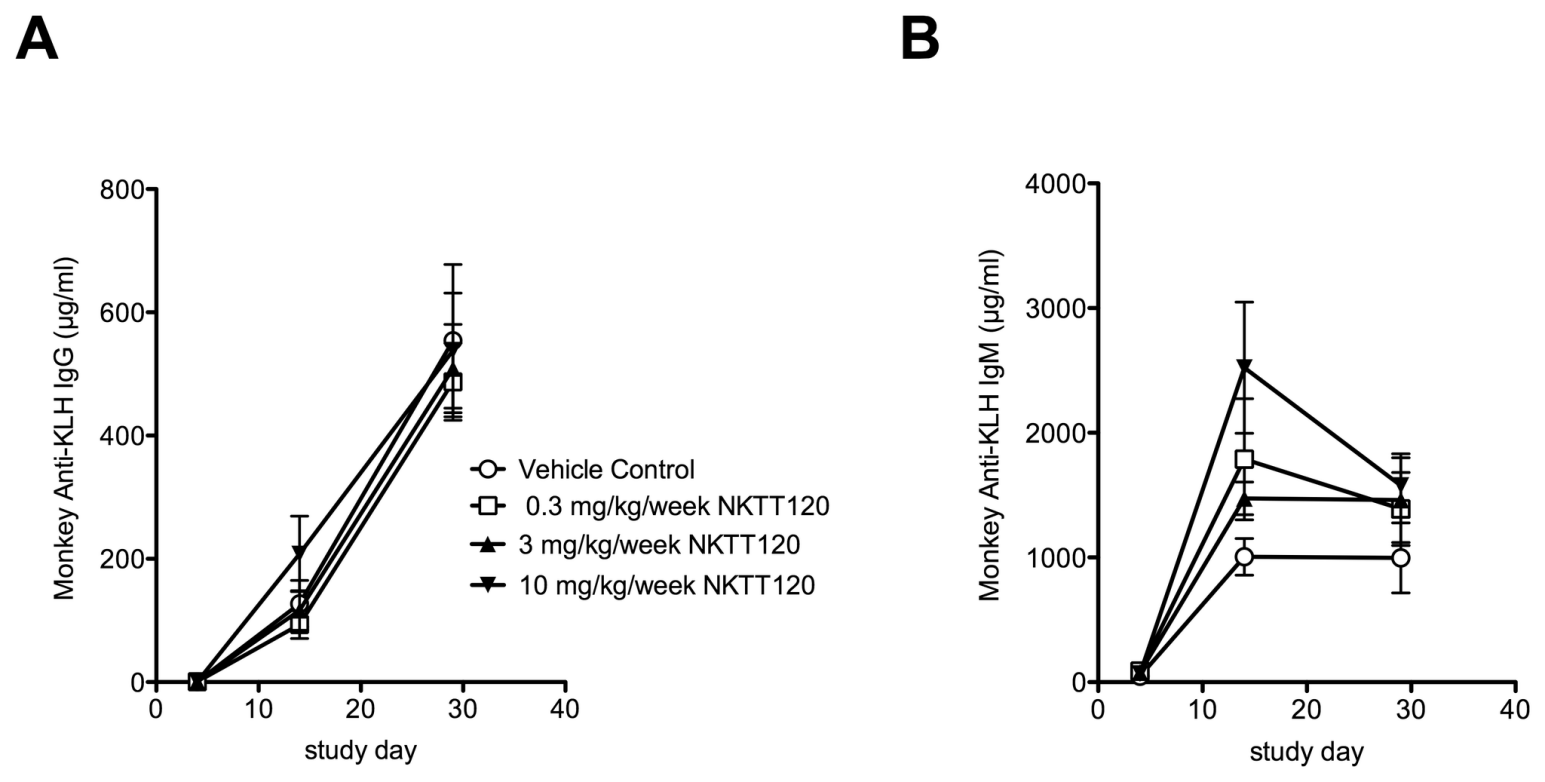
NKTT120 was administered to cynomolgus macaques (3-5/sex/group) at 0, 0.3, 3, and 10 mg/kg/week via intravenous injection once weekly for 5 doses. Keyhole Limped Hemocyanin (KLH) (3 mg/mL, at a dose volume of 1 mL/animal) was administered subcutaneously between the shoulder blades of the monkeys on Study Days 4 and 19. Blood (processed to serum) for anti-KLH IgM and IgG measurements was collected on Study Days 4 (prior to KLH administration), 14, and 29; anti-KLH IgG (**A**) and IgM (**B**) antibody response was measured by ELISA. One way ANOVA with multiple comparison was performed using PRISM software. There was no significant difference in Antibody responses across all groups.

To test if the pharmacologic depletion of iNKT cells will suppress T cell mediated antibody responses, we evaluated the response to KLH immunization in this study. The immune response to KLH challenge was not attenuated by iNKT cell depletion. The IgM response seemed to be higher in the NKTT120 dose groups, while the IgG response to KLH immunization was comparable between the dose groups and control (Figure **7**). To further determine if iNKT cell depletion can be done safely, we sought to determine if NKTT120 binding to the iTCR induces an inflammatory cytokine release by iNKT cells prior to their depletion. To that end serum cytokines (IL-1β, IL-4, IL-6, IL-10, TNF-α, and IFN-γ) that are associated with iNKT cell activation were measured after each dose. Cytokine levels in all of the treatment groups were below the level of quantification at all time points. The only test article-related microscopic finding was in the thymus; at all dose levels a reduction in cortical lymphocytes was noted. However, after the eight week recovery period, there were no microscopic findings in thymuses from control animals or animals previously given 10 mg/kg/week. Overall, no adverse events were observed in any of the dose groups at any time point.

## Discussion

A series of in vitro and in vivo studies have been performed as part of the nonclinical profiling of NKTT120 to support its evaluation in human clinical studies. Collectively, these studies show that NKTT120 can potently, selectively, and safely bind to and deplete iNKT cells and that iNKT cells can recover in peripheral blood in a concentration- and time-dependent manner. NKTT120 binds only to human and old world non-human primates’ (cynomolgus macaques, rhesus macaques) iNKT cells. The specificity of binding to iNKT cells via the iTCR was further demonstrated by evaluating the binding to 9 human derived cell lines. None of these cells express the iTCR and none bound NKTT120. As an IgG1 antibody, the mechanism of cell depletion would be expected to be via antibody and/or complement mediated cytotoxicity [14]. The Biacore binding assays found that the primary affinity of NKTT120 was for Fc receptors and not for C1q, which demonstrated a low affinity for NKTT120. This suggests that the primary mechanism of iNKT cell depletion would be expected to occur through ADCC. This was confirmed in the in vitro ADCC and CDC assays where ADCC mediated killing of cells expressing the human iTCR occurred at concentrations of NKTT120 that were 2 logs lower than that required for equivalent CDC mediated cell killing. However, despite the high affinity of NKTT120 for the human and monkey iTCR, following NKTT120 dosing of non-human primates, iNKT cells did not exhibit a pattern of activation, as indicated by the absence of elevated cytokine levels or signs of target organ injury, as had been observed in murine models with other activators of iNKT cells, such as α-galactosylceramide [15]. Absence of cytokine release was also observed in unpublished studies of in vitro activation of human blood by NKTT120. The absence of an activation profile is most likely the result of the rapid iNKT cell depletion mediated by NKTT120 or it may be related to the low activation status of the normal monkeys used in this study. However supporting the former hypothesis, in our recently initiated clinical studies with NKTT120 in sickle cell patients with activated iNKT cells, we have also seen no indication of a cytokine release response at doses that produced a reduction in human iNKT cells (unpublished observations). The slightly elevated IgM and comparable IgG response to KLH immunization vs. control animals suggests that the T cell mediated immune response was not negatively impacted by iNKT cell depletion.

Pharmacokinetic parameters were assessed in the cynomolgus monkey following a single intravenous dose. The data from this study showed that the t_½_ for NKTT120 is approximately 17.5 days which, along with other measured PK parameters, are consistent with those of other therapeutic monoclonal antibodies [16]. This long half-life suggests that dosing regimens could be structured to manipulate iNKT cell numbers for short or long term depletion. This dose related impact on iNKT cells was observed in the two non-human primate studies reported here. In these studies, doses ranged from a single dose of 0.01, 0.03, 0.1 or 0.3 mg/kg to 5x repeat doses of 0.3 mg/kg, 3 mg/kg and 10 mg/kg. In pilot studies that are not reported here, a dose of 0.003 mg/kg resulted in a partial (50%) and transient reduction (~4 days) of iNKT cells. Doses < 0.003 mg/kg did not affect peripheral blood iNKT numbers. As would be expected, the high dose repeat administration of NKTT120 resulted in a complete depletion with no restoration of measurable peripheral blood iNKT cells over the course of the 28 (0.3 and 3 mg/kg) or 56 (10 mg/kg) days monitored in this study. Although the single dose administration of NKTT120 study is somewhat limited by the fact that each group consisted of 3 animals, the overall trend in this study suggests that recovery of iNKT cells was dose and plasma concentration dependent. The 0.01 mg/kg dose group had measurable iNKT cells at weeks 2-3 when plasma concentrations reached 60 ng/mL and reached the 0.03% level within 5 weeks when plasma concentrations of NKTT120 were ~30 ng/mL. The 0.03 mg/kg group had iNKT cells return following a similar plasma concentration relationship at 4 and 7 weeks respectively. The two higher dose groups had recovery responses that were more variable. In the 0.1 mg/kg dose group the blood iNKT cells were measurable at week 7 for one and week 10 for the other two monkeys. Only one animal reached 0.03% for the remainder of the monitoring period while the other two animals had iNKT cell counts that were just below or above 0.03% from week to week. In the 0.3 mg/kg group the initial appearance of iNKT cells in blood was observed at week 7 for one animal and week 15-16 for the other two monkeys. The more variable responses at the higher doses may be related to the extent of tissue iNKT cell depletion produced by the various doses administered to the monkeys confounded by the small number of animals in each group. There was no obvious reason for the early response of the one monkey at week 7 in the 0.3 mg/kg group. Our unpublished observations are that the dose required to significantly deplete total tissue iNKT cells is approximately 10-fold that necessary to produce total blood depletion of iNKT cells. The long time required for recovery of five of the six animals in the 0.1 and 0.3 mg/kg dose groups would support this observation. The 0.01 and 0.03 mg/kg doses depleted whole blood iNKT cells but both doses demonstrated a recovery of all animals that correlated with plasma concentrations in the range of 30-60 ng/mL of NKTT120. At 10-fold higher concentrations the recoveries were much more variable and, with the exception of one animal, the plasma concentrations were below the 30-60 ng/mL concentrations that were required to maintain blood iNKT cell depletion by ADCC. This is consistent with a broader tissue depletion following these higher doses that requires much more time to stimulate regeneration of iNKT cell regeneration from primitive precursor cells. This observation on a slower recovery at higher doses would also be consistent with the observation that doses of 0.3 mg/kg or higher in our repeat dose study resulted in reversible thymic hypocellularity, suggesting both iNKT depletion and a possible impact of this depletion on other cell populations in the thymus. iNKT cells develop in the thymus, similar to other T cells, and represent a clonal population that share the same invariant TCR-α, a uniquely rearranged Vα24-Jα18 (Vα14-Jα18 in mice). Studies in mice show that iNKT cells, unlike conventional T cells, acquire a memory phenotype during their natural development by recognizing endogenous antigens presented on CD1d molecules on CD4, CD8 double positive thymocytes in the thymus without requiring prior exposure to foreign or pathogenic antigens. Due to their memory phenotype, they can be rapidly activated and expand within the peripheral immune compartment in response to either endogenous glycolipid antigens or exposure to foreign glycolipid antigens presented by APCs. Thus, for the reconstitution of iNKT cells after depletion there are two sources of iNKT cells: continuously generated new emigrants from the thymic compartment and cells already within the periphery which have the capacity to rapidly expand in response to exposure to antigen or immunological insults. Our data are consistent with this dual source of reconstitution hypothesis. The lower doses (0.01 and 0.03 mg/kg) depleted peripheral iNKT cells and possibly some tissue cells and recovered by expanding from tissue iNKT cells once plasma concentration of NKTT120 reached 30-60 ng/ml. The two higher doses (0.1 and 0.3 mg/kg) had recoveries that were more variable and consistent with a greater depletion of tissue iNKT cells and reseeding from thymic emigrants rather than tissue iNKT cells, as indicated by a lag in recovery when plasma concentration of NKTT120 reached the 30-60 ng/ml concentration associated with recovery of the lower doses of NKTT120. Clonal loss or diminution of memory T cells, or creation of a "hole" within the immune repertoire for a given individual, is a well-known phenomenon that has been attributed to pan-lymphocyte- or T cell-depleting antibodies that are in current clinical use, or in clinical development [17]. In humans, iNKT cell regeneration has been studied following bone marrow transplantation. The recovery of iNKT cell numbers occurs in weeks to months following transplantation [18–20]. Thus, unlike conventional T cells, the continuously regenerative capacity of iNKT cells obviates similar long-term concerns with pharmacological intervention using iNKT-depleting mAbs. The data presented in this study further supports the concept that iNKT cells can be transiently depleted by NKTT120 for a period of time for therapeutic purpose followed by a slow and sustained recovery.

Recently, iNKT cell activation has been demonstrated in sickle cell disease and it has been suggested that depletion of these cells could have benefit reduction of the thrombo-inflammatory state found in these patients [9–12]. The overall response to NKTT120 administration in nonhuman primates suggests that it would be a safe and effective way to modulate iNKT cells in the sickle patient as a means to evaluate the role of these cells in the pathophysiology of sickle cell disease. In this regard, we have recently initiated a clinical trial in stable sickle cell disease patients to test this hypothesis (ClinicalTrials.gov Identifier: NCT01783691)
